# Inclusive governance: Enhance health literacy to reduce vaccine hesitancy

**DOI:** 10.1093/eurpub/ckac129.738

**Published:** 2022-10-25

**Authors:** V Bjegovic-Mikanovic

**Affiliations:** Institute of Social Medicine, Faculty of Medicine, University of Belgrade, Belgrade, Serbia

## Abstract

**Background:**

The COVID-19 pandemic placed a call for action worldwide. Based on scientific investigation, governments need to assess strategic priorities. Health system capacity constraints and failures in response to the pandemic have social, medical, productivity, and economic implications. It compounds health equity issues and confronts with excess mortality, higher chronic disease prevalence, and risk factors. Despite initial progress in vaccination against COVID-19 and attempts to speed up vaccination, the Western Balkan lags behind. Infodemic and low trust in institutions are among the main factors associated with low success and adverse effects on other vaccination programs. This presentation aims to shed light on the importance of health literacy in resilient communities supported by inclusive governance.

**Methods:**

A narrative review based on literature on inclusive governance, health literacy, and resilient communities during COVID-19 and other emergencies. Primary sources are databases, scientific articles, Health System Response Monitor, and observations by ECDC and OECD.

**Results:**

Results show that characteristics of resilient communities in possessing knowledge and ability to assess risk, manage an emergency, monitor change, and address threats stand out. Many studies highlight the interconnectedness of community members with the wider external environment and their participation in decision-making to improve health services. Examples include interventions for developing future vaccination programs in program planning, conducting sound evaluations, transferring results to those who need to know, and receiving feedback. The key to this success is enhancing digital and health literacy.

**Conclusions:**

COVID-19 requires cross-sectional strategies to reinforce collaborative gains and build resilient communities, ready to apply population strategies for prevention. Inclusive governance and a bottom-up approach will be essential to optimize the response to future challenges.

